# Piloting a Shared Source Water Treatment Intervention among Elementary Schools in Bangladesh

**DOI:** 10.4269/ajtmh.18-0984

**Published:** 2019-09-23

**Authors:** Farzana Yeasmin, Farhana Sultana, Leanne Unicomb, Fosiul Alam Nizame, Mahbubur Rahman, Humayun Kabir, Peter J. Winch, Stephen P. Luby

**Affiliations:** 1 International Center for Diarrheal Diseases Research, Bangladesh, Dhaka, Bangladesh;; 2 Johns Hopkins Bloomberg School of Public Health, Baltimore, Maryland;; 3 Stanford University, Stanford, California

## Abstract

Hundreds of thousands of children continue to die each year from diarrhea. We piloted a low-cost liquid chlorine point-of-use (POU) water treatment among elementary school children in Bangladesh. We began the 1-month intervention in four schools (two urban and two rural) by introducing POU drinking water hardware and behavior change communication. We trained teachers to deliver sessions encouraging students to drink chlorinated water from their own small plastic bottles to avoid disease transmission. We used cue cards and flip charts as visual aids. We evaluated the acceptability, feasibility, and potential for sustainability after 1 month and after 14 months of the intervention. During 1-month follow-up, among 141 drinking events observed, 141 students (100%) drank chlorinated water. In 93 or 66% of events, students used their own bottles, and in 43 (30%) of the events, they used common cups or hands washed before drinking. During the 14-month follow-up, we observed 732 drinking events. In 653 of 732 events (89%), students drank chlorinated water; in 78 events (11%), they consumed water from untreated drinking water sources. Among those who consumed chlorinated water, 20% (131/653) used their own bottles to drink water, 72% (467/653) used common cups, and 8% (55/653) used both hands to drink water. Most stated that they drank chlorinated water because it is safe, it has health benefits, and treatment reduces germs. Introduction of specific hardware, weekly hygiene sessions, and education materials enabled schools to treat water at POU and students to consume treated water.

## INTRODUCTION

Although the number of diarrheal deaths has reduced over recent decades, hundreds of thousands of children continue to die each year from diarrhea.^1^ Point-of-use (POU) water treatment with chlorine reduces reported diarrheal disease.^2,3^ Chlorine is a low-cost accessible water treatment option.^4^

Most studies on promotion of point-of-use (POU) water treatment have focused on households.^5^ These studies have documented consistently low uptake, with the exception of some studies on boiling.^6^ A key difficulty has been the need to convince each individual household to adopt and use the POU technology. In Dhaka, Bangladesh, low uptake of four low-cost household POU products was noted even among households who received products free of cost and repeated educational messages about the importance of drinking safe water.^7^ Moreover, there was a low willingness to pay for these products.^7^ Household water treatment requires individual-level time, effort, and new habit adoption within each household, which might contribute to low chlorine-based POU technology uptake.^3,8^

Although schools potentially offer more favorable conditions for implementing POU water treatment, there are few studies from this setting.^9^ Water, sanitation and hygiene (WASH) programs in schools can improve health, dignity, and comfort for students and teachers.^10^ Schools are an important place of learning and have existing organizational, social, and communication structures, which provide opportunities for health education and a health-enhancing environment.^11^ A positive school environment can provide an opportunity to create life-long changes in health behavior,^11^ and in later life as parents, these children can teach better hygiene habits to their own children.

The school setting allows for more intensive delivery of the intervention and for creation of a favorable environment (enabling environment) for behavior change.^11^ In a study in rural Kenyan primary schools, water treatment with hypochlorite increased in 17 rural primary schools.^12^ In this study, the fieldworkers provided flocculent-disinfectant powder and hypochlorite solution for water treatment to the school. They trained the teachers and the students how to install water stations and provided instructional comic books. Comic books which were distributed to all students described how to treat water using water treatment products. Students were encouraged to read it, discuss it in the class, and even take it home to show to their parents. Free sachets of a water treatment product were also given to the students to take home and demonstrate to their parents.^12^ In addition, parents were aware of this POU method even 12 months after the school-based promotion ended.^12^ In Kenya, the rates of diarrhea decreased by more than half among students exposed to a chlorine-based drinking water intervention in schools.^13^

In contrast to the low uptake found in Bangladeshi households where each individual household decided whether to chlorinate water, we explored how a more centralized decision in an institutional setting would affect water treatment uptake with the potential to impact a larger number of people. This article reports the results of a pilot study exploring local interest in POU water treatment for elementary school students and teachers in urban and rural Bangladesh, and a follow-up to determine schools’ abilities to fund recurrent costs.

## METHODS

### Study setting and participants.

We enrolled total eight elementary schools: four rural schools in Mymensingh district and four in Dhaka (the capital city of Bangladesh) using purposeful selection to cover a variety of contexts. We selected four schools (two urban from Dhaka and two rural from Mymensingh) for the formative phase of the study and four schools (two urban from Dhaka and two rural from Mymensingh) for the subsequent intervention pilot phase. The school selection criteria were as follows: 1) located in low-income communities, 2) government and nongovernment, 3) coeducation with student population ≥ 250, 4) no participation in a WASH program within the previous 2 years, and 5) drinking water supply tested and found to be contaminated with *Escherichia coli*.

### Study phases.

**Figure 1.**
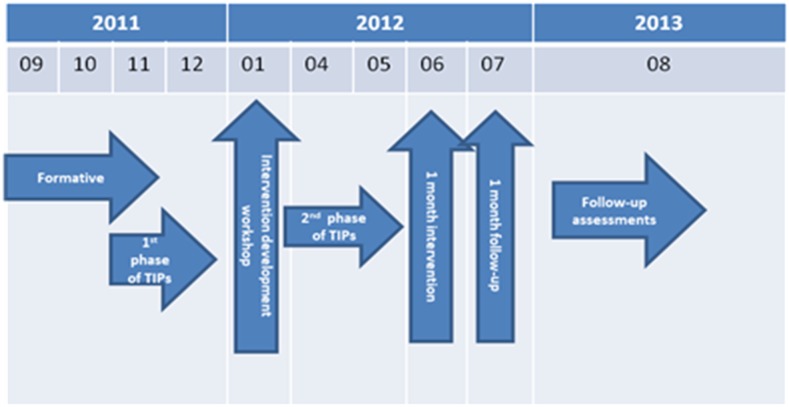
Study timeline. This figure appears in color at www.ajtmh.org.

Total duration of the study was 16 months from May 2011 to September 2012. We conducted the formative research from September to November 2011 to identify and develop an integrated set of interventions (hardware and behavior change communication). During the subsequent pilot intervention phase, we conducted a 1-month intervention and 1-month follow-up from June to July 2012. We then conducted follow-up assessments 14 months postintervention in August 2013 to examine sustained adoption of the promoted water treatment technology in intervention schools (Figure 1).

### Baseline data collection methods.

From May 2011 to November 2013, the field team used several data collection methods, including surveys, in-depth interviews, and focus group discussions to fulfill different objectives. They also conducted trials of improved practices (TIPs) for the hardware.^14^ We conducted formative research in schools to collect information on current drinking water practices, perceptions on the safe water storage hardware options provided, willingness to participate in POU water treatment, and their opinion on feasibility. We structured our qualitative interview guides using the Integrated Behavioral Model for Water, Sanitation and Hygiene (IBM-WASH) framework to explore influential behavioral factors at contextual, psychosocial, and technological levels.^15^

### Student and head teacher survey.

We collected information from the students of grade IV and V and head teachers using structured questionnaires. Trained fieldworkers conducted the survey with 50 students and one head teacher in each of four schools. For student surveys, we randomly selected 25 students from all sections of each grade from a list we prepared from the class register books. We recorded students’ demographic information, students’ knowledge of WASH, diarrhea and respiratory diseases, and students’ and head teachers’ suggestions for effective channels of communication related to WASH interventions.^16^

### In-depth interviews.

Trained fieldworkers conducted 20 in-depth interviews with head and science teachers, school management committee members, and parent–teacher association members and an engineer from the local government engineering department, focusing on perceptions of hygiene-related topics and potential WASH interventions among the schools in the formative phase. We conducted interviews until we reached saturation.

### Focus group discussions.

Fieldworkers conducted 12 focus group discussions with students, assistant teachers, and school management committee and parent–teacher association members using separate guidelines to assess WASH knowledge and perceptions, preferred hardware options, communication channels and accountability methods.

### Trials of improved practices for the hardware.

We analyzed data from the formative phase and shared the findings through an intervention development workshop.^16^ We convened a 1-day intervention development workshop held in January 2012 at icddr,b. Workshop participants included teachers, school management committee members, parent–teacher association members, and members of the study team. We shared findings from the formative phase and sought feedback for acceptability and feasibility to chlorine-treated water for drinking. We developed a water treatment intervention to address the reported barriers related to POU water treatment methods for safe drinking water. The field team conducted TIPs^14^ in four schools in the formative phase in Dhaka and Mymensingh to aid development of a water treatment intervention. Trials of improved practices comprised two phases: first, we provided hardware (liquid chlorine dispenser and initially a 100-L and then a 60-L water storage tank with a tap based on school feedback) and water treatment products (Aquatab^™^ [Aquatabs^®^, Medentech, Wexford, Ireland] and liquid chlorine) and collected feedback from students and teachers. Subsequently, we made feedback-based improvements. Students used the hardware, and we collected further feedback. Fieldworkers formed school hygiene committees composed of students, teachers, education officials, janitors, and school management committee and parent–teacher association members to maintain hardware, treat water, and raise funds for chlorine. It was a completely new role and subject area for the participants.

### Chlorine purchase and dilution.

As part of purchasing 5.5% chlorine and laboratory costs for dilution, we expended US$ 24.3 for 8 L of 2.1% liquid sodium hypochlorite (bleach). All schools were provided one bottle of 2-L dilute bleach that cost US$ 6. The school management committee members, parent–teacher association members, and the teachers of the hygiene committee of all schools paid the cost after the intervention was completed. We applied a uniform dose of liquid sodium hypochlorite, which our earlier studies suggested effective, in reaching the WHO-targeted free chlorine residual across a wide range of available source water,^17^ rather than customizing the dose to each individual source of water.

### Intervention.

Summarized formative data were used to guide design and implementation of the intervention. Fieldworkers promoted the intervention, including hardware and education materials and assessed the acceptability and feasibility of the intervention. Treated water was not the only source available during the intervention. Students had access to both the treated water in the tanks and untreated water from the original source. The four intervention schools received the hardware and 2 L of 2.1% liquid chlorine; this was sufficient for 6 months. Fieldworkers suggested that before the chlorine finished, the school community could request additional supplies by phoning them. The school community called the fieldworkers before the chlorine finished, and the fieldworkers provided 2 L of 2.1% liquid chlorine to the schools for another 6 months.

Cue cards and laminated flip charts were used to communicate the behavioral recommendations as part of the behavior change plan. Cue cards described the steps of using the chlorine dispenser. Both the cue cards and the flip charts were primarily visual. The school teachers were trained at the International Center for Diarrheal Disease Research (icddr,b) to lead behavior change sessions using pictorial flip charts and cue cards in conjunction with the regular weekly hygiene classes. In the training sessions, the teachers were trained to lead the behavior change sessions where teachers were asked to encourage students to drink safe water. In the pictorial chart topic, the teachers described how the students identify unsafe water, why it is not good for their health, which diseases can occur by drinking unsafe water. They also introduced chlorine dispenser, the benefits of using chlorine dispenser. The school staff were not trained to adjust chlorine dose.

**Figure 2.**
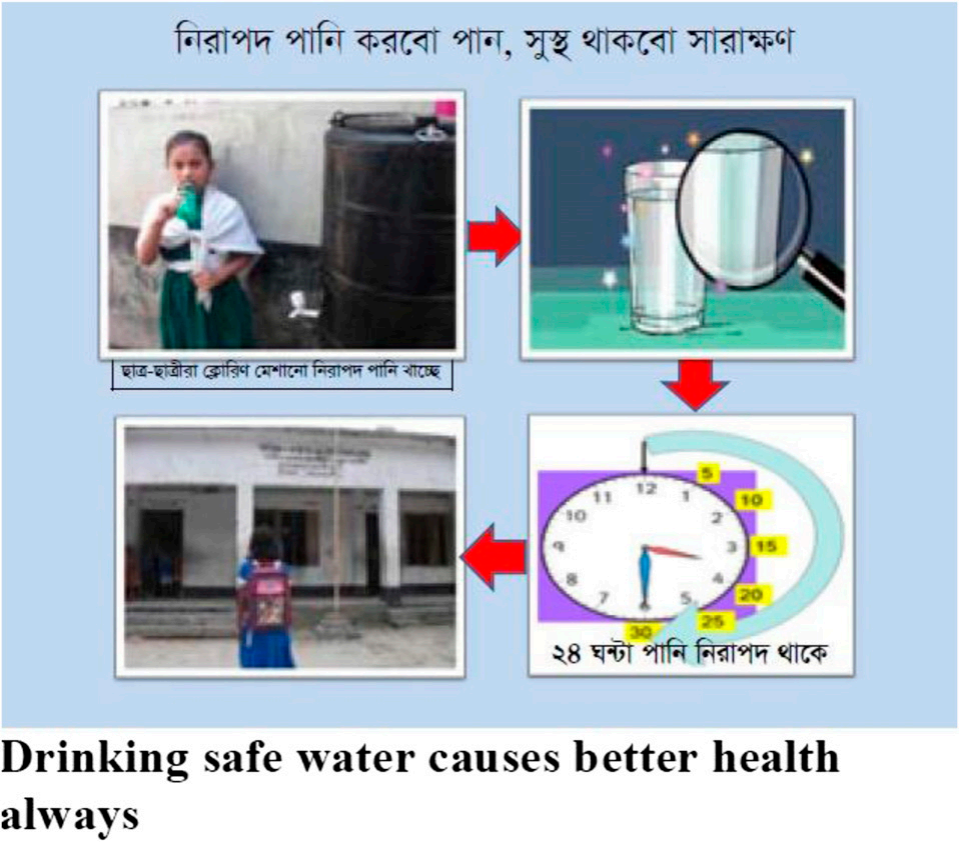
The cue card attached on school walls. This figure appears in color at www.ajtmh.org.

The pictorial chart also encouraged the students to bring their own bottle and fill that with chlorinated water to drink safe water. The intervention also supported that if the students could not bring their own bottle then they should use the communal cups to drink chlorinated water. The cue cards were mainly the replication of the flip charts that were provided to be attached on school walls and next to the hardware to call students for action toward proper practice. We developed 11.69 × 16.54-inch cue cards depicting the recommended behaviors to fix next to the hardware and classrooms. The cue cards included the following: ensuring half-an-hour dosage after chlorination and before drinking and use of own bottle for drinking water (Figure 2). Fieldworkers implemented the interventions in four different schools for 1 month from June to July 2012. The trained teachers continued the interventions after the first 1-month intervention promoted by the fieldworkers.

### Postintervention data collection.

#### One month postintervention.

Fieldworkers conducted four-hour structured observations (on six occasions, days 1, 2, 3, 7, 14, and 30 after the first class on drinking chlorinated water) in each school (total 24 observations) for students’ drinking behaviors. During lunch break, the fieldworkers located from where both treated water and untreated water source could be observed and conducted structured observations on students of all ages. Using structured guidelines, fieldworkers conducted informal discussions with teachers and students to collect feedback on hardware and behavior change communication materials to revise accordingly. The fieldworkers also conducted a qualitative assessment to explore the acceptability, feasibility, use, and potential for sustainability of the POU intervention. The participants of the focus group discussions and in-depth interviews were mainly students of grade IV and V who were aged 8–13 years (Table 1). Fieldworkers conducted 34 focus group discussions with grade IV and V students (promoted behavior adopters and nonadopters), head and assistant teachers, school management committee members, and parent–teacher association members in each of the four schools. Each focus group discussion had five to eight participants. Fieldworkers conducted four pocket voting exercises where 24 male and female students from both grade IV and V were randomly selected from each of the four intervention schools to participate.

**Table 1:** Summary of data collection methods

Phase	Method	Main objective(s)	Data collected
Pre-pilot	Formative study: teacher survey, in-depth interviews (*n* = 20), and focus group discussions (*n* = 12);	To explore knowledge, perceptions, reported practices, and barriers to drink safe water	Demographic characteristics of respondents, existing practices of drinking water, and problem of drinking safe water
	trials of improved practices (*n* = 2)		
Trials of improved practices (pilot)	Pretested the hardware	To explore knowledge, perceptions, to identify problems in drinking chlorinated water by using chlorine dispenser, and maintain the hardware	Key feedback on hardware modification and sustainability
	Hardware provision, BCCs, forming hygiene committee, and train the teachers of hygiene committee	To train the teachers of hygiene committee for continuation the intervention	Knowledge and practice of safe drinking water and maintenance of hardware
Post-pilot (1-month follow-up assessment)	Structured observations: 6 in each school (*n* = 24), focus group discussions (*n* = 34) and in-depth interviews (*n* = 4)	To explore knowledge, perceptions, reported practices, and barriers to drink safe water by using chlorine dispenser;	Benefits and barriers to drink safe water by using chlorine dispenser, observed use of drinking water hardware
		to observe the practice of drinking water	
Post-pilot (14-month follow-up assessment)	Structured observations: 3 in each school (*n* = 12), focus group discussions (*n* = 22)	To explore knowledge, perceptions, reported practices, and barriers to drink safe water by using chlorine dispenser;	Benefits and barriers to drink safe water by using chlorine dispenser, observed use of drinking water hardware
		to observe the practice of drinking water	

#### Fourteen months postintervention.

To assess sustainability of recommended behaviors, fieldworkers conducted an unannounced follow-up visit 14 months after the intervention in each school. They conducted 12 structured observations of student drinking behaviors in each intervention school on three consecutive school days using the same approach as the 30-day assessment. Fieldworkers also conducted a quick survey in 1 day to know the self-reported drinking chlorinated water information. The qualitative assessment took the whole month. They conducted 22 focus group discussions with students classified as promoted behavior adopters and nonadopters based on structured observation, head and assistant teachers, members of the school management committee, and parent–teacher association members in each of the four intervention schools. Each focus group had six to eight selected participants who were available, and student participants were mainly from grades IV and V. The fieldworkers conducted a spot check in each school for the presence of water in the storage tanks and structured observations of student drinking behaviors. The field team assessed ongoing use of the intervention by testing for residual chlorine using a Hach Chlorine Test Kit (Total Chlorine Color Disc Test Kit, Model CN-66T; Hach Company, Loveland, Colorado); the color wheel was used to visually match the color in the test vial to a numerical free or total chlorine reading. The test kit measured chlorine in the range of 0–3.5 mg/L, equivalent to 0–3.5 ppm (parts per million). We assessed sustainability in two ways: 1) uptake (observed drinking events) and 2) user’s perceptions and experiences of using the water from shared treated sources to assess the feasibility and acceptability.

### Data analysis.

The focus group discussions and in-depth interview data were recorded and for the qualitative data analysis, the audio-recorded data were transcribed and then translated into English. The data were collected by six experienced researchers with academic backgrounds in either anthropology or other social science. The data were analyzed by the authors. We applied a priori codes, based on the research objectives and the components of the IBM-WASH framework to code the data.^15^ We created codes to refer to behavioral factors associated with drinking chlorine-treated water along the contextual, psychosocial, and technological dimensions in the IBM-WASH framework. After coding, we summarized the major themes and the findings for each of the predefined codes. Descriptive analyses were performed followed by cross-tabulations to calculate frequencies. Fieldworkers did not record students’ gender during the structured observations and so did not explore gender differences in behavior.

### Ethics.

We took permission from the Divisional Primary Education Office of the Government of Bangladesh to conduct our study in schools from Dhaka and Mymensingh districts. We obtained teachers’ written consent, and students assented before conducting data collection. This study was approved by icddr,b Ethical Review Committee.

## FINDINGS

### Baseline.

Among the four formative study schools (two urban and two rural), half of the urban school students reported that the water they drank at school was boiled water, which they brought from their home. All rural school students reported that they drank untreated drinking water from available sources on the school premises (Table 2). Teachers from urban schools said they had previously suggested that students carry boiled water from home in their own bottles because there were no arrangements for safe drinking water within the school compound (Table 2). Urban school teachers reported that the students were mostly from low-income communities (Tables 3 and 4) and had limited access to boiled water from home or money to purchase filtered water. The major barrier at urban school compounds was the absence of arrangements for provision of safe drinking water by school authorities. Before the intervention, personnel in the rural schools perceived that untreated drinking water sources were 100% safe for drinking. Water treatment methods with chlorine and provision of water storage vessels were perceived as potentially helping students secure safe drinking water in school compounds. During the baseline survey, few students (15%) reported that transmission of diarrhea could occur from drinking unsafe water.

**Table 2:** The type of existing water source in the formative schools, pre-pilot perceptions on safe drinking water and drinking water practices among students

	Urban schools (on the premises)	Rural schools (on the premises)
Water source	Municipal supplied (piped)	Untreated drinking water sources
	Untreated drinking water sources	
Perceptions of school water source	Considered school water unsafe for drinking unless boiled or filtered where piped water	Considered school water safe for drinking
	Considered school water safe for drinking where untreated drinking water sources	
Practices related to drinking water at school	Most students drank the piped water	All students drank water from the untreated drinking water sources using common cups or their hands
	Some brought boiled water from home in their own bottles and few purchased filtered water from shops	

**Table 3:** Sociodemographic characteristics of study respondents in urban Dhaka and rural Mymensingh

Type	Formative study, *n* (%)	Fourteen-month follow-up assessment, *n* (%)
Student	248 (89)	31 (63)
Education		
Grade IV	124 (45)	5 (9)
Grade V	124 (44)	26 (49)
Occupation of the students guardian		
Farmers	49 (20)	2 (6)
Salaried government job	39 (16)	2 (6)
Salaried nongovernment job	–	3 (10)
Small trader	36 (15)	4 (13)
Small business	32 (13)	12 (39)
Nonagriculture labor	27 (11)	3 (10)
Van/rickshaw operator	23 (9)	–
Other	42 (17)	4 (13)

**Table 4:** Sociodemographic characteristics of study respondents (teacher) in urban Dhaka and rural Mymensingh

Type	Formative study, *n* (%)	Fourteen-month follow-up assessment, *n* (%)
Teachers and school management committee members	30 (11)	22 (45)
Education		
Elementary	6 (2)	6 (11)
Secondary	9 (3)	0
Higher secondary	7 (3)	8 (15)
Graduation	8 (3)	8 (15)
Monthly income of teachers and school management committee members in US$		
No income	3 (10)	4 (18)
63–125	11 (37)	9 (41)
126–188	10 (30)	7 (32)
Above 189	7 (23)	2 (9)

### Trials of improved practices for hardware and treatment products.

Most of the students reported that liquid chlorine (sodium hypochlorite)-treated water smelled less and tasted better than Aquatab-treated water. Teachers reported that because guidelines stated that water was safe for drinking for up to 48 hours after treatment, they discarded chlorinated water every day and so did not need to move heavy water tanks inside at the end of the day (Table 1).

One female grade IV student at an urban school said: “*There is no smell (in the liquid chlorine treated water) but the tablet had a bad smell and this (liquid chlorine treated) water tastes good.*”

All the teachers reported that the lack of a specific person to clean, store, and maintain the 100-L and 60-L storage tank was a major barrier. The tap on the provided tanks was difficult to turn off, so water dripped and made the school compound wet and dirty.

**Figure 3.**
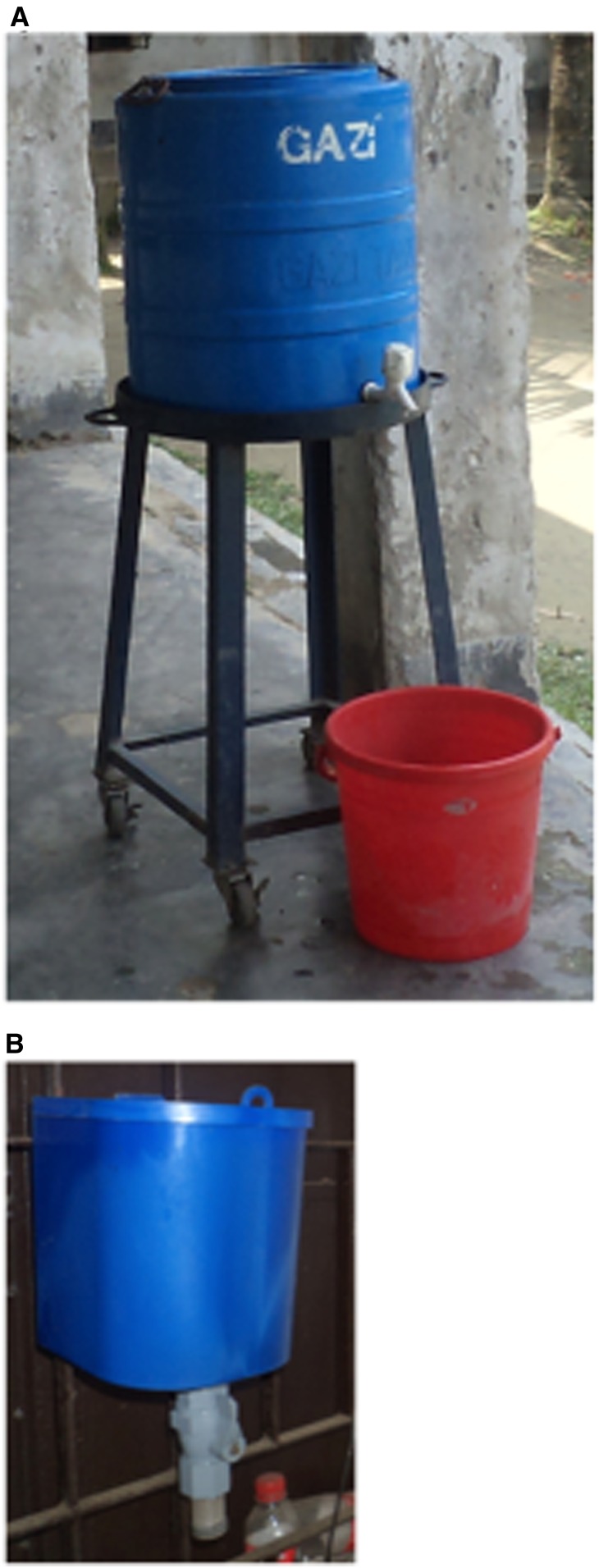
The hardware provided for the drinking water treatment intervention: (**A**) 60-L storage tank, stand, and bucket (100-L tank not shown); (**B**) chlorine dispenser. This figure appears in color at www.ajtmh.org.

The final versions of the hardware (Table 5), selected for implementation in the four intervention schools, after feedback from TIPs included a 100-L tank with handles, tank stand, and lock (Figure 3). The intervention water treatment product was liquid sodium hypochlorite with a dispenser^17^ (Figure 3). Head teachers of all schools were responsible for treating water with liquid chlorine. To create a supportive physical environment, in addition to providing the hardware preferred by the school community, we ensured that schools installed and identified means for maintaining the hardware. The fieldworkers instructed the hygiene committee members to add 12 mL (four turns) of liquid chlorine, by regulating the dispenser knob manually, to the 100-L water tank and wait for half an hour. They advised that students could drink treated water for up to 24 hours. We addressed the structural component of the IBM-WASH model by working with school hygiene committees to either review or develop rules and regulations for drinking water and then put them in place to promote recommended behaviors (Table 6).

**Table 5:** The hardware design and behavioral change communication materials provided to intervention schools for promoted behaviors and the recommended practices

Promoted behaviors	Hardware design for urban school	Hardware design for rural school
POU for urban and rural school	100-L tank with handles	100-L tank with handles
	60-L water storage tank with tap	60-L water storage vessel with tap
	a pipe for filling water in the vessel, metal stools	a jar (*kolshi*) to fill the vessel with water
	2 L of 2% liquid chlorine for six months use with a dispenser and a lock	flattened bar stools with wheels
		2 L of 2% liquid chlorine with a dispenser and a lock
BCC materials for both urban and rural schools	11.69 × 16.54-inch cue cards and weekly basis sessions conducted by teachers using laminated flip charts	11.69 × 16.54-inch cue cards and weekly basis sessions conducted by teachers using laminated flip charts
Recommended practice for both urban and rural schools	Drinking chlorinated water using their own small plastic bottles (to avoid probable transmission of communicable diseases)	Drinking chlorinated water using their own small plastic bottles (to avoid probable transmission of communicable diseases)

**Table 6:** Summary of key findings using the IBM-water, sanitation and hygiene model^17^

Level of influence	Implications for intervention design
*Contextual-level barriers*	
Access: Lack of availability of liquid chlorine in local shops	Continued promotion and availability of liquid chlorine in local shops could support and improve safe drinking water availability at schools
*Contextual-level benefits*	
Favorable environment for habit formation: School environment was feasible for this intervention	Schools may provide supportive environment for adoption of water chlorination.
School hygiene committee played a positive role to continue the intervention	School hygiene committee can be developed and can play a big role
*Psychosocial-level barriers*	
Taste and smell: taste and smell of chlorinated water seems bad	The message includes “smell could ensure that the water is safe” would work
*Psychosocial-level benefits*	
Existing habits: most of the students were motivated to drink chlorinated water. They perceived it as a medicine	Children perceived it as a medicine, which makes water germ free, which is a positive perception
*Psychosocial-level facilitators*	
Shared values: school community had a strong shared value for chlorinated water and worked for maintenance	School hygiene committee should be emphasized for developing shared value in the school community which will include collective efficacy for maintenance
*Technological-level barriers*	
Convenience: in rural area, students are not accustomed to carry their own bottles as it not so common there	Future behavior change communication materials and methods should emphasize the need to use individual bottles to reduce transmission via shared cups or dirty hands
Strengths and weaknesses of the hardware: although chlorine dispensers were well accepted, some teachers expressed difficulty maintaining supply of chlorinated water using the storage tanks	The message, including that for motivating teachers, students, and janitors, would be helpful. The maintenance system would consider the proper timing which will not hinder in class time

### One-month follow-up.

During focus group discussions, all the teachers, students, and school management committee and parent–teacher association members stated that liquid chlorine was an acceptable product for making drinking water safe for schools because it was easy to treat water by dosing using the chlorine dispenser (Figure 3B). They felt that chlorine continuously killed germs and made water safe to drink for 24 hours. Urban students said that drinking chlorinated water released them from the burden of carrying boiled water from home, and rural students said that it freed them from pumping untreated drinking water sources existing on the school premises or having to go to neighbors’ houses for water while at school.

One female student from grade V at an urban school said: “*If we drink this (chlorinated) water we can come to school regularly, can concentrate on study and so we can have good results in the examination.*”

Most students found the cue cards placed near the drinking water point good motivators for drinking chlorinated water. Some teachers and students found the provided cue cards useful for safe drinking water promotion. Most of the students reported that chlorinated water smells bad but still they continued drinking treated water because they considered it safe and good for health.

All four intervention schools successfully formed hygiene committees that included of students, teachers, education officials, janitors, and school management committee and parent teacher–association members. These committees were charged with maintaining the hardware and raising funds to cover the cost of the dilute bleach. Formation of hygiene committee institutionalized the intervention promotion for the school setting. Teachers were responsible for treating the water. During the 1-month implementation phase, fieldworkers instructed the hygiene committee members how to add 12 mL (four turns) of liquid chlorine, by regulating the dispenser knob manually, to the 100-L water tank and wait for half an hour. They advised that students could drink the treated water for up to 24 hours.

**Figure 4.**
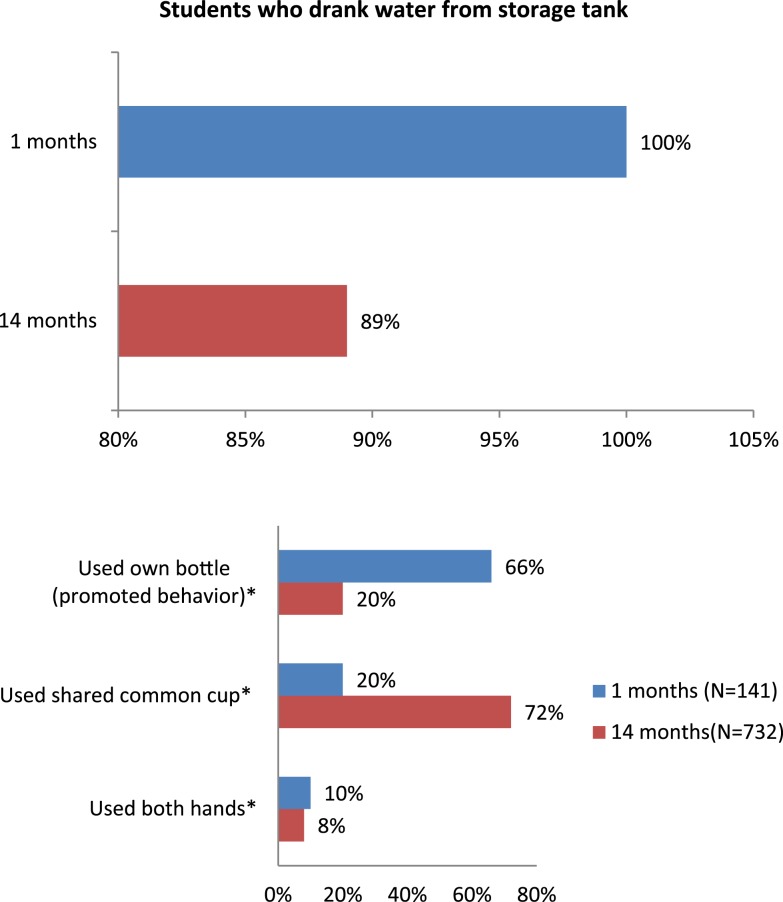
Observed student drinking behaviors at 1-month and 14-month intervention follow-up periods. This figure appears in color at www.ajtmh.org.

In 1-month assessment, fieldworkers observed 141 occasions of students drinking water; in all, 141 (100%) students drank chlorinated water. In 93 or 66% of events, students used their own bottles, and in 43 (30%) of events, they used common cups or hands washed before drinking (Figure 4).

### Fourteen-month follow-up.

Fieldworkers confirmed the use of 2% chlorine to treat water at all schools, as the presence of residual chlorine was between 0.2 mg/L and 4 mg/L at urban schools and 0.8 at both rural schools. The school management committee members, parent–teacher association members, and the teachers of the hygiene committee of all schools paid the cost of chlorine after their supply was used (US$6.00/2 L bottle of liquid chlorine). During the 14-month follow-up, fieldworkers observed 732 drinking events. In 653 of 732 events (89%), students drank chlorinated water, and in 78 events (11%), they consumed (untreated) water from untreated drinking water sources. In 131 or 20% of chlorinated water drinking events, students used their own bottles, in 467 (72%) of events, they used common cups, and in 55 (8%) of events, they used hands washed before drinking (Figure 4). We did not observe any differences in behavior from 1 day to the next.

The adopter students at both urban and rural schools perceived chlorine as a medicine that treats water, helps to kill germs, and, therefore, makes water safe to drink.

One male student from grade V at a rural school said: “*We feel well when we drink chlorinated water because we know it is safe for our health.*”

Most students at rural schools either used common cups or both hands to drink chlorinated water because they did not carry drinking water bottles to school.

One urban school teacher reported that visitors from other elementary schools appreciated the introduction of liquid chlorine to provide safe drinking water and became interested to purchase liquid chlorine to treat drinking water at their schools. Respondents that participated in the study commonly mentioned that the safe drinking water intervention worked very well in the schools because the whole school community perceived it as essential. They reported that either the janitors or the student members of the hygiene committee cleaned the water storage vessels, refilled water, and treated it with liquid chlorine. Students from the rural schools’ hygiene committee were very willing to perform this function. During the 14-month follow-up visit, all 1,133 students present in 1 day reported drinking chlorinated water (100%); 18 (1.6%) said they disliked the taste and smell of chlorine but drank treated water for health benefits. All schools covered the cost of chlorine provided by icddr,b fieldworkers after the initial 2 L was depleted.

### Perceived barriers to drinking treated water.

Some teachers reported difficulty in maintaining a sufficient supply of chlorinated water using the provided storage tanks. One teacher at an urban school said: “*Water becomes finished within the lunch period that we often need to refill again, dose with chlorine and wait for half an hour. Though we have two vessels (one 60 L and one 100 L), it is difficult to maintain because we don’t have enough space to place them.*” A few students complained that maintaining hardware sometimes made them miss part of their classes.

Nonadopter students mentioned that both the water tank and chlorinated water becomes hot during summer season; therefore, they preferred drinking filtered or untreated drinking water sources that is colder. Some nonadopter students from urban schools mentioned that their mothers perceived boiled water as safe and provided them a bottle of boiled water.

Some students mentioned that the water storage vessel looked unclean. Students at one urban school mentioned that the chlorinated water storage vessel was placed on the ground floor, which served as a barrier for the students who attended classes on the second and third floors.

Most of the students recommended placing larger attractive posters on school walls. Some students recommended that the school provide clean cups or bottles for increased convenience. One girl from an urban school said: “*I never share my bottle with others because it is unhealthy and transmits diseases from one to another.*”

## DISCUSSION

We observed remarkably high uptake and acceptability of a low-cost liquid chlorine drinking water treatment even up to 14 months after a brief, 1-month intervention in schools. This stands in contrast to the experience with continuous and longer term promotion of household-level chlorination where the chlorine-based interventions have been taken up by < 10% of households, despite providing repeated educational messages about the importance of drinking safe water.^17^ Factors favoring higher uptake and acceptability in this study include the centralized leadership, control, and decision-making in the school environment.^11^ The intervention created an enabling environment supporting safe drinking water that was absent before the intervention. Following focused efforts to explain the intervention, school leaders and teachers understood the potential benefits of the intervention and supported it. Also, centralized water treatment in schools meant that a smaller group of decision makers needed to be persuaded to treat their water in contrast to householders who need to be persuaded one by one. By contrast, in one study, although there was a steady supply of chlorine with repeat visits by field research assistants, there was limited commitment among urban slum household members to treat their water with chlorine,^7^ highlighting the advantage of centralized decision-making on water treatment.

Schools in Bangladesh do not always provide safe drinking water for students. In a national population-based assessment of water facilities, the majority of schools had improved and functional drinking water sources (80%).^18^ However, 13% of school students carried drinking water from home rather than using the school sources,^18^ and improved sources can be contaminated with fecal bacteria.^19^ At the structural level, we determined that schools had the commitment, human resources, and financial resources to maintain equipment and provide a steady supply of chlorine to create an environment conducive for treating drinking water. However, schools in our intervention did not need to purchase the hardware.

The intervention built a favorable environment for water treatment habit formation. The school hygiene committee members played a supportive role in management of the chlorination process. In a school-based intervention study in Kenya, the teachers were trained on behavior change and water treatment methods and there were follow-up visits throughout the school year. They found significant differences in WASH knowledge between intervention and control groups. Intervention schools showed significant improvement in drinking chlorinated water.^9^ In households, a supportive environment for adoption of water chlorination is absent when no one takes a leadership role.^7^

At the individual level, we observed that students developed a habit of drinking chlorinated water and sustained that habit over time, evident from structured observations 14 months after intervention delivery. This contrasts with the global experience in household settings, wherewith substantial resource commitment and uptake remained quite low, especially among the lowest income households whose children are at greater risk of death from contaminated water.^6^ In a recent study conducted in low-income urban compounds in slum communities in Dhaka where multiple households share common cooking areas, toilets, and water collection points, residents disliked the medicinal smell of chlorinated water but became accustomed to it during the study.^4^ In the same study, participants also mentioned that they collected and stored chlorinated drinking water because they thought it was safe for their children. A likely explanation for sustained water treatment in our study, even when schools had to pay for chlorine themselves, is that individual behavior change was convenient; neither each student nor each family had to treat their water individually as this was performed centrally. We did not examine whether children influence the behavior of their families in this study.

In this study, the most commonly cited barriers were taste or smell and wait time, barriers that have been noted previously.^8^ In school, the regulated environment and peer pressure likely helped to overcome barriers such as taste or smell. Schools treated water at the end of school time and so the smell was likely reduced when students drank water the next day. The school hygiene committee members, especially students and teachers, were role models and motivated those who disliked the smell and taste. People from Bangladesh dislike the smell and taste of chlorinated water,^7^ and so when they are choosing individually, most do not treat. By contrast, when it is not a matter of individual decision-making, initial nonadopters can become convinced of the value of treatment and learn to tolerate smell/taste.

A major limitation to scaling up the findings of this study was that high-quality properly diluted sodium hypochlorite was an essential element of the intervention, but the product is not generally available in the local market; this would need to be made available for the intervention to be adopted at scale. Sodium hypochlorite that is available in Bangladesh is not of a consistent concentration and quality to use for dosing drinking water. Some organizations recommended treating household water with low-cost, widely available commercial bleach to improve water quality, but with varying concentrations in commercial products, it is difficult to consistently achieve concentrations that are effective and not too malodorous.^20^ An appropriate dosage regime developed by testing each batch of commercial bleach for sodium hypochlorite concentration and calculating the appropriate dose for that batch^20^ is not practical. The World Health Organization’s guidelines for emergency water treatment suggest adding known quantities of each batch of commercial bleach to source waters until the correct residual is found.^21^ For either method, trained operators are needed to perform ongoing water quality testing and ensure an appropriate dosage regime for each batch of commercial product for a consistent effective program. Expecting sufficient expertise at each school to conduct chlorine testing and adjust dosage is unrealistic, although expertise at the subdistrict level might be more feasible. This expertise might be provided by the Department of Public Health Engineering in Bangladesh, who have laboratories across the country and may provide support not only to schools but other institutions including hospitals.

We collected data from only four rural and four urban intervention schools among a small number of participants. Assessments were limited by the small sample size. We intentionally included schools from both urban and rural settings. The overall environment of the enrolled schools was like others in Bangladesh, and we attempted to include a range of elementary school types (e.g., schools with deep water, shallow water and piped water supply). In addition, because no other water, sanitation, or hygiene programs took place in these schools for 6 months prior or during the study period, we expect that the findings are typical of what we would expect in the broader school community.

Another limitation on the potential effectiveness of our intervention was the use of shared communal cups. Although we encouraged school children to bring their own bottle from home, many children in rural communities found this difficult as bottles were not readily available in their households. Engineering an option that would elevate the water container and permit a fountain dispenser would avoid the need for a cup and so reduce the risk of person-to-person transmission.

The chlorine dispenser intervention in a school setting had much greater acceptability than household-level chlorination in urban Bangladesh. Moreover, we found sustained water treatment practice, supported by schools developing their own funding strategies. Future school interventions should include long-term strategies to reliably provide essential, reliable supplies of quality-assured chlorine and make it locally available.
